# Impaired Reproductive Performance of Waterbirds in Metal-Contaminated Tropical Rice Agroecosystems: Evidence from Little Egrets (*Egretta garzetta*)

**DOI:** 10.3390/toxics13080676

**Published:** 2025-08-13

**Authors:** Hanxun Qiu, Xin Huang, Chuanbiao Xu, Jiliang Zhang

**Affiliations:** 1 Ministry of Education Key Laboratory for Ecology of Tropical Islands, College of Life Sciences, Hainan Normal University, Haikou 571158, China; 910281@hainnu.edu.cn (H.Q.); 202312071300023@hainnu.edu.cn (X.H.); 400040@hainnu.edu.cn (C.X.); 2 Hainan Provincial Key Laboratory of Ecological Civilization and Integrated Land-Sea Development, Hainan Normal University, Haikou 571158, China

**Keywords:** heavy metals, agroecosystems, waterbirds, little egrets, breeding performances, exposure risk, biomonitoring

## Abstract

Heavy metal pollution in rice fields is a major concern; however, little research has addressed its exposure and risk to waterbirds inhabiting rice fields. We investigated the accumulation of heavy metals (Cd, Pb, As, Cr, Cu, and Zn) in sediment, water, food, feces, feathers, and eggshell samples collected from different nesting sites (Chongwei Village and Wuji Village) of little egrets (*Egretta garzetta*) on Hainan Island, China, and compared the differences in their breeding parameters and eggshell quality. Higher levels of heavy metals were observed in all samples except feces from Wuji Village compared to those from Chongwei Village. As, Cd, and Pb exhibited little bioaccumulation in all feather and eggshell samples, whereas Cr concentrations in feather samples from both heronries and eggshell samples in Wuji Village exceeded the toxicity threshold in birds, indicating that the high maternal Cr was transferred to eggs in Wuji Village. Significantly lower hatching and breeding success rates were observed in Wuji Village than in Chongwei Village, which may be closely related to Cr contamination. This study revealed that waterbirds breeding in rice fields are under threat from heavy metal contamination and highlighted the suitability of bird feathers and eggshells as biomonitors of the environment.

## 1. Introduction

The emission and legacy of heavy metals are major global issues that threaten biodiversity and the health and quality of ecosystems owing to their challenging environmental degradation, bioaccumulation, and potential toxicity [1]. Agricultural activities are one of the main contributors to the introduction of these pollutants into the environment [2]. Previous studies have shown that intensive agriculture, characterized by the extensive use of fertilizers and pesticides, has led to alarming levels of heavy metals in the environment [2,3]. Rice fields are one of the most frequent places subjected to intensive agricultural practices and cover 160 million hectares of land in 112 countries worldwide [4]. In addition to providing an important food supply for humans, they are home to numerous wildlife species, including waterbirds, and play an important role in protecting and increasing biodiversity, particularly as natural habitats continue to decline worldwide [4,5]. Therefore, the extensive accumulation of heavy metals in rice field agroecosystems and their potential ecological consequences are of great concern.

Environmental pollution is often assessed using biological sentinel species that best represent environmental quality and health [6,7]. Because of their high position in the food chain and sensitivity to environmental changes, birds are considered the most suitable sentinels and are extensively used as indicators of environmental pollution [8,9]. For environmental risk assessment, previous studies have evaluated heavy metal exposure risk to birds in wetland ecosystems using risk models based on the contamination levels of metals in the environment (sediment and water) and/or food items [10,11,12]. However, this assessment method may be questionable because some heavy metals, although present at high levels in sediment, water, and/or lower trophic organisms, may exhibit no or low bioaccumulation in bird tissues [13].

The potential risks and effects of heavy metals on birds depend on the extent of heavy metal accumulation. Therefore, the measurement of heavy metals in various bird tissues is important because it can provide a better understanding of the extent and impact of metal pollution. Recently, the use of a variety of bird tissues, such as feathers, eggshells, blood, muscle, liver, and kidneys, has been reported in exposure risk studies of environmental metal pollution [7,14,15,16]. However, the scope of use of different tissues is sometimes limited. For example, the collection of internal tissues by sacrificing birds is highly invasive and has been criticized, limiting its suitability for studies involving endangered or rare species. Therefore, the use of different tissues for risk assessment should be carefully considered based on the study’s scope. Feathers and eggshells have been used extensively as non-invasive matrices to evaluate heavy metal contamination in birds [14,17,18]. Feathers can serve as useful indicators of toxic metals deposited in the blood and body [19,20]. Metal contamination in eggshells can represent egg contamination, which is associated with the risk of exposure to laying birds and offspring [17,21]. Therefore, analyses of heavy metals in feathers and eggshells can provide valuable information on the risks posed to the health of adult birds and nestlings.

Recently, a growing number of studies have suggested that in addition to continuous monitoring of heavy metal concentrations in bird tissues, more emphasis should be placed on survival probabilities and other life history traits, such as reproductive performance, to fully reveal the potential effects of environmental heavy metal pollution on free-living birds [22,23,24,25]. Environmental metal pollution affects bird breeding. Previous studies have shown that environmental metal pollution has negative effects on different aspects of reproduction, including an increased frequency of egg-laying interruptions, changes in egg characteristics (such as egg size and eggshell thickness), reductions in clutch size, and decreased hatching and fledging success [21,23,24,25,26,27]. However, previous studies on the reproductive risks of environmental metals in bird populations have primarily focused on passerine birds, such as tree sparrows (*Passer montanus*), russet sparrows (*Passer rutilans*), and blue tits (*Cyanistes caeruleus*) breeding in artificial nest boxes deliberately placed in areas with heavy mining or industrial contamination. However, the validity of the results obtained from artificial nest boxes as a proxy for what happens in wild nests may be subject to question [22]. To the best of our knowledge, few studies have focused on the reproductive risks of waterbirds living in other heavily non-point source polluted areas, such as rice field agroecosystems, although 38% of waterbirds are suffering from population decline worldwide, largely due to environmental pollution [11].

The little egret (*Egretta garzetta*), a top predator that primarily feeds on fish, is at high risk of environmental metal exposure and is considered a species of concern in ecological risk assessments [28,29]. Rice fields are important foraging and breeding habitats. In China, Hainan Island has several nesting colonies and is one of the most important breeding areas for little egrets [29]. Although Hainan Island has less industrial activity, the excessive use of chemical fertilizers and pesticides has led to serious enrichment of heavy metals in its arable land soils because of the high utilization rate of tropical agricultural lands [30,31]. Therefore, the risk of environmental metal exposure to waterbirds in tropical rice fields is of particular concern. Arsenic (As), cadmium (Cd), chromium (Cr), lead (Pb), copper (Cu), and zinc (Zn) are heavy metals of particular concern in agricultural areas. Therefore, in this study, we investigated the accumulation of these heavy metals in foraging environments, food/prey items, feces, feathers, and eggshell samples of little egrets from different nesting sites on Hainan Island. Furthermore, we investigated the differences in breeding parameters (clutch size, hatching success, fledging success, and breeding success) and eggshell quality (eggshell thickness) between the different nesting sites. This study aimed to systematically reveal the potential ecological risk of heavy metal pollution in rice field agroecosystems to the reproduction and health of resident waterbirds. It is hypothesized that the breeding parameters and eggshell quality of little egrets are lower at nesting sites with a higher exposure risk of heavy metals.

## 2. Materials and Methods

### 2.1. Study Area

Hainan Island, located in the northern part of the South China Sea, is one of the most important tropical islands and the second-largest island in China. It covers an area of 35.4 × 10^3^ km^2^ and has a tropical ocean monsoon climate, with an annual mean precipitation of 1685 mm and a temperature of 23.8 °C [32]. Hainan has few industrial activities and is known for its agriculture, with arable land accounting for more than 20% of its total area, which provides important winter vegetables and tropical agricultural products in China. Rice is the staple crop of Hainan, and its extensive cultivation provides an important habitat for various waterbirds, including little egrets. The use of chemical fertilizers and pesticides has increased significantly in recent decades owing to the lower fertility and higher utilization rate of arable land in Hainan [30]. For example, the annual consumption of chemical fertilizers increased from 8.63 × 10^5^ t in 2006 to 1.18 × 10^6^ t in 2010 [33]. It reached a peak of 1.36 × 10^6^ t in 2015 and then gradually declined as the government began to take measures to reduce the use of chemical fertilizers in 2016 [34]. However, the long-term overuse of chemical fertilizers and pesticides has led to the introduction of large amounts of heavy metals into the arable land of Hainan [30,31].

**Figure 1:**
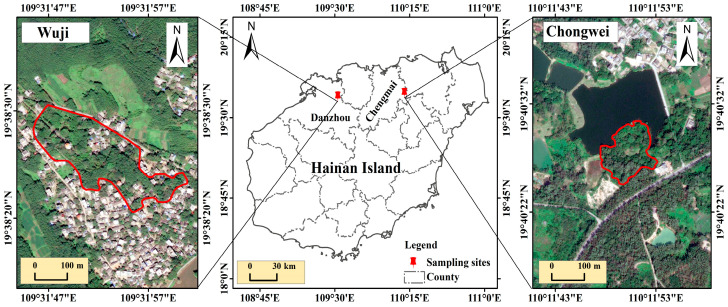
Geographical location of the sampling sites in Hainan, China. The area surrounded by the red line represents the nesting area of the little egret.

Hainan is an important breeding habitat for egrets in China owing to its unique conditions. Chongwei Village and Wuji Village are the most important breeding sites for little egrets in Hainan [29]. They are 70 km apart and are located in the Chengmai and Danzhou districts of Hainan Island (Figure 1). The little egrets at the two sites mainly utilize the surrounding rice fields as their habitats for foraging and breeding. Little egrets in Chongwei Village mainly nest in the bamboo forest, while in Wuji Village, they mainly nest on banyan trees. A previous study found a higher anthropogenic input of heavy metals in arable land soils in the western part of Hainan [30]. Therefore, little egrets in Wuji Village may suffer from more serious threats posed by heavy metal pollution than those in Chongwei Village.

### 2.2. Breeding Monitoring

We monitored bird breeding performance at both nesting sites during the breeding season (April to August 2023). First, we randomly selected and marked 72 and 95 nests of little egrets with numbered flags in the Chongwei Village and Wuji Village heronries, respectively. We then monitored nest contents weekly until the chicks could leave the nest and move onto the tree branches near the nest (“fledglings” hereafter). Thus, it was easy to determine the number of eggs laid, nestlings, and fledglings per nest. We obtained reproductive information on (1) clutch size (maximum number of eggs observed during the visit), (2) hatching success (eggs hatched/eggs laid), (3) fledging success (fledglings/eggs hatched), and (4) breeding success (fledglings/eggs laid). These metrics cover multiple endpoints at different stages of the reproductive cycle and have been widely investigated in reproductive risk assessment studies [35]. During the monitoring period, if a nest and/or all nest contents suddenly disappeared from one visit to the next, except for the departure of fledglings, the nest was considered destroyed. All destroyed nests were omitted from the reproductive metric analyses.

### 2.3. Sample Collection

During breeding monitoring, we collected sediment and water samples from the surrounding foraging areas, post-hatching eggshells, feathers, and feces samples of adult little egrets, as well as their food items in both nesting sites.

According to a previously reported method [28], sediment samples were collected from the surrounding foraging habitats within 10 km^2^ of the breeding grounds. Briefly, five sub-samples (approximately 2.5 kg) were collected from a depth of 0–5 cm in approximately 1 acre of foraging area using a plastic shovel to combine them into a composite sample, and ten replicates were taken from each heronry. Sediment samples were stored in aluminum bags and brought back to the laboratory for analysis. Approximately 10 L of the surface water layer was collected in a plastic bucket in the same area and thoroughly mixed. Subsequently, approximately 500 mL of the composite water was collected in an acid-washed glass bottle, acidified with 1 mL concentrated HNO_3_ (65%), and kept at 4 °C [10]. Ten composite water samples were collected from each heronry.

According to a previously reported method [36], fresh feathers and feces of adult little egrets were randomly collected from different marked nests and trees before hatching. Five sub-samples (five feathers or five pieces of feces) were pooled to form a composite sample, and ten composite feather and feces samples from different nests were collected from each heronry. Collected feather and feces samples were stored in aluminum bags and plastic Petri dishes at −20 °C before analysis, respectively. Similarly, fresh post-hatching eggshells were randomly collected from different marked nests and beneath the trees and kept in aluminum bags at −20 °C before analysis. In total, 59 and 62 eggshell samples were collected from the heronries of Chongwei Village and Wuji Village, respectively. Food samples were collected according to previously reported methods [13,28]. Briefly, fresh food samples were collected via the spontaneous regurgitation of nestlings. They were randomly taken from beneath trees (on which marked nests were built) and preserved in labeled aluminum bags at −20 °C before analysis. Ten food samples were collected for each heronry.

### 2.4. Laboratory Analysis, Quality Assurance, and Control

Sediment samples were dried at 60 °C in an oven and thoroughly ground using a ceramic mortar and pestle to pass through a 100-mesh sieve. Preprocessed sediment samples (0.5 g) were weighed and digested with 12 mL of *Aqua regia* (HNO_3_:HCl = 3:1) in Teflon vessels using an intelligent digestion system (PreeKem TOPEX+, Shanghai, China) [11]. The digestion procedure consisted of two stages: a heating phase at 200 °C for 15 min and a reaction phase at 200 °C for 40 min.

Eggshell samples were used to determine eggshell thickness and heavy metal content. According to previously reported methods [21,27], eggshells were first cleaned using cotton swabs dipped in 95% ethanol and dried at room temperature. The thickness of the eggshells was then determined latitudinally to the nearest 0.01 mm. After determining eggshell thickness, the samples were washed three times with deionized water and an acetone solution (1 mol/L). Eggshells were then dried at 60 °C and ground into powder. Preprocessed samples (0.5 g) were removed and placed in Teflon vessels for digestion with 10 mL of concentrated HNO_3_ (65%), in ten replicates for each heronry. The digestion procedure was the same as described above.

According to a previously reported method [28], food samples were washed with tap water and deionized water, subsequently dried at 60 °C, and ground using a porcelain mortar. Feather samples were washed successively with tap water, deionized water, and acetone solution (1 mol/L); subsequently dried at 60 °C; and dissected into small pieces [20,23]. Feces samples were dried directly in an oven at 60 °C and homogenized using a porcelain mortar [37]. Preprocessed food, feathers, and fecal samples (0.5 g) were transferred to Teflon vessels and digested with 10 mL of concentrated HNO_3_ using the intelligent digestion system. The digestion procedure was the same as described above.

The digestion solutions obtained above were evaporated at 105 °C to around 1 mL. After cooling, the solution was quantitatively transferred to a volumetric flask, diluted to 25 mL with deionized water, and filtered through a 0.45 μm membrane filter. Water samples were directly filtered through a 0.45 μm membrane filter before measurement. The concentrations of six metals (As, Cd, Cr, Pb, Cu, and Zn) in all digests and water samples were measured using an inductively coupled plasma mass spectrometer (Agilent 7900, Santa Clara, CA, USA). Before the beginning of each digestion, all utensils, including Teflon vessels used in the experiment, were immersed in a HNO_3_ solution (15%, *v*/*v*) for 24 h, rinsed three times alternately with tap water and deionized water, and subsequently dried at 60 °C in an oven to prevent potential external metal contamination and cross-contamination. Furthermore, to verify and ensure the accuracy of the measurement process, blanks and certified reference standard materials with known concentrations were used in the same manner. The sediment standard material (GBW07407) from the National Research Center for Geoanalysis of China and the biological standard material (GBW08517) from the Second Institute of Oceanography of the Chinese State Oceanic Administration were used to verify the measurement accuracy [38,39]. A mixed calibration standard of Cd, Pb, Cr, As, Cu, and Zn (10 μg/mL, Agilent, Part# 5183-4688) was diluted to produce a standard solution series with HNO_3_ (5%, *v*/*v*). The standard curves were automatically drawn by the instrument, and their correlation coefficients were >0.999. The limits of detection (LOD) were 0.003, 0.002, 0.004, 0.008, 0.005, and 0.01 μg/L for Cd, Pb, Cr, As, Cu, and Zn, respectively. The limits of quantification (LOQ) were 0.01, 0.007, 0.01, 0.03, 0.02, and 0.04 μg/L for Cd, Pb, Cr, As, Cu, and Zn, respectively. The recovery rates (measured value/standard reference value × 100%) of the studied metals ranged from 94 to 108%.

### 2.5. Calculation and Statistical Analysis

Bioaccumulation factors (BAFs) for biological matrices were calculated according to the concentrations of heavy metals in the biological matrix (*C_b_*, μg/g dwt) and in the sediment of the foraging environment (*C_s_*, μg/g dwt) using the following equation [17]:

BAF = *C_b_*/*C_s_* (1)

Biomagnification factors (BMFs) for bird tissues were calculated according to the concentrations of heavy metals in the bird tissues (*C_bird_*, μg/g dwt) and in the food (*C_food_*, μg/g dwt) using the following equation [40]:

BMF = *C_bird_*/*C_food_* (2)

IBM SPSS (version 22.0) was used for the statistical analysis. Heavy metal concentrations in sediment, food, feces, feather, and eggshell samples were presented as μg/g dry weight (μg/g dwt), while the concentrations in water samples were presented as μg/L. All data were expressed as mean ± SD. Shapiro–Wilk and Levene tests were adopted to check the normality of the data and homogeneity of variance, respectively. An independent-sample *t*-test was used to compare the differences in heavy metal concentrations in different samples between the two heronries, as the data were normally distributed. The Mann–Whitney U-test was used to compare any differences in breeding parameters and eggshell thickness between the two heronries, as the data were not normally distributed [24]. For all statistical tests, *p* < 0.05 was considered significant. Graphs and sampling location maps were generated using Origin version 2024 and ArcGIS version 10.8.

## 3. Results and Discussion

### 3.1. Heavy Metal Contamination in Foraging Habitats

The concentrations of heavy metals in sediment and water samples from the foraging habitats of both little egret heronries are presented in Table 1. In the sediment samples, the concentrations of As, Cd, and Cr in Wuji Village and the Cd concentration in Chongwei Village exceeded the Chinese environmental background values for soil and the threshold effect level of Canadian Freshwater Sediment Quality Guidelines. However, the concentrations of all heavy metals in the water samples from both heronries were lower than the class I standards of the Chinese Environmental Quality Standards for surface water and the permissible limits suggested by WHO guidelines for drinking water. The higher concentrations of heavy metals in the sediment than in the water are consistent with the findings of previous studies [10,12]. This phenomenon may be related to the low water solubility of heavy metals and easy adsorption by sediments [12]. Additionally, adsorption by aquatic plants may also lead to low concentrations in water [41].

Notably, the Cd and Cr contents in the sediment from Wuji Village were 14-fold and 1.6-fold higher than the background levels, respectively, and the value of Cd in the sediment from Chongwei Village was 7 times higher. This suggests that Cd and Cr are the main heavy metal contaminants in the foraging areas of little egret heronries on Hainan Island. The levels of these heavy metals were higher in this study than in the foraging areas of little egret heronries in other regions of China, such as Poyang Lake, Tai Lake, and the Pearl River Delta [13], as well as the Jabbi area of Pakistan [44]. Compared with islands in other territories, the Cd and Cr concentrations in the sediment were higher than those on Fisherman Island and in Western Moreton Bay of Australia [45], but the Cd concentration was lower than that in the Baja California Peninsula of Mexico [46] and Ivujivik in Canada [47]. Although Hainan Island has less industrial activity, the large amounts of fertilizer and pesticide used in various agroecosystems have resulted in high levels of heavy metals (especially Cd and As) in the soil relative to other provinces of China because of the higher utilization rate of agricultural land and lower soil fertility [31]. Except for Cd in water samples, the concentrations of all heavy metals were significantly higher in the sediment and water samples from Wuji Village than in those from Chongwei Village (*p* < 0.05) (Table 1). This result is in accordance with the work of Wang (2015) [30], who found a greater anthropogenic input from heavy metals, such as Cd and As, in arable land soil in the western part of Hainan (where Wuji Village is located). Our results suggest that Cd, As, and Cr are the main toxic metal pollutants in rice field agroecosystems in Hainan and may pose threats to waterbirds and their biodiversity.

### 3.2. Heavy Metals in Food and Feces of Little Egrets

**Figure 2:**
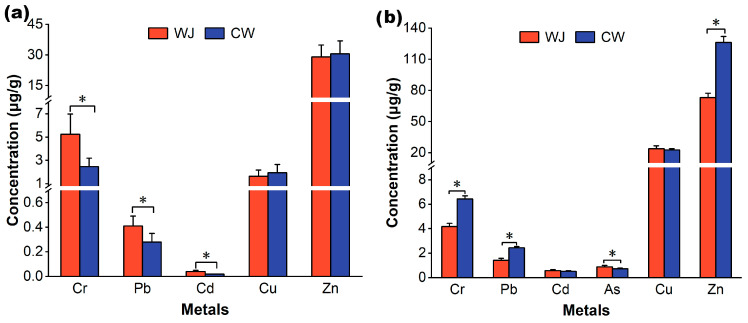
Comparison of metal concentrations measured in food (**a**) and fecal (**b**) samples of little egrets from Wuji (WJ) Village and Chongwei (CW) Village heronries. * Significant differences between the two heronries.

A summary of the concentrations of the studied heavy metals in food samples of little egrets from the Wuji Village and Chongwei Village heronries is presented in Figure 2a. As arsenic was not detected in any food samples, it was not included in Figure 2a. Similarly, previous studies have found high concentrations of As in the foraging habitats of little egrets but not in their food items [13,48]. Unlike the foraging habitats of little egrets (Table 1), the general trend of heavy metal accumulation in the food items of both heronries was Zn > Cr > Cu > Pb > Cd. Compared with other metals, the BAFs for Zn and Cu in food items were the highest (Table 2). Furthermore, the concentrations of Cr, Pb, and Cd were significantly higher in food samples from the Wuji Village heronry than in those from the Chongwei Village heronry (*p* < 0.05), whereas the Zn and Cu contents were not significantly different between the two heronries (*p* > 0.05) (Figure 2a). These results suggest that Zn and Cu have high bioaccumulation potential in food samples relative to Pb, Cd, and As, which is consistent with previous findings [13]. Zn and Cu are essential elements that play vital physiological roles in regulating biochemical reactions; thus, organisms can regulate the quantities of these elements to a stable level through homeostatic mechanisms [7,21].

The concentrations of all heavy metals detected in fecal samples of little egrets from the Wuji Village and Chongwei Village heronries are presented in Figure 2b. Although As was not detected in their food samples, a high concentration of As was detected in fecal samples from the Wuji Village and Chongwei Village heronries, with mean concentrations of 0.87 and 0.71 μg/g, respectively. All studied metals in fecal samples showed high levels of accumulation, with the highest BAFs (Table 2). Compared to their food samples, higher concentrations of all heavy metals were found in the fecal samples from both heronries (Figure 2). A previous study similarly found that fecal samples of the American dipper (*Cinclus mexicanus*) from the Chilliwack watershed in British Columbia, Canada, contained higher concentrations of heavy metals than their food items [49]. Bird feces often reflect metal contamination in bird foraging environments and diets [50]. Previous studies have suggested that birds ingest toxic metals from food, sediment, and water, with the former considered the main source [12,51]. However, our results suggest that for toxic metals with low bioaccumulation in food, such as As, Cd, and Pb, contaminated sediment and water in foraging habitats may represent the primary exposure routes for little egrets. Similarly, a previous study found that the levels of As, Cd, and Pb ingested by gray herons (*Ardea cinerea*) living in the Mai Po Inner Deep Bay area, China, from the sediment and water accounted for 73.5%, 77.8%, and 94.9% of their total intake, respectively [11]. These results emphasize the significance of direct ingestion of toxic metals from the foraging environment in waterbirds.

Notably, unlike the foraging habitats and food samples of little egrets, the concentrations of most of the studied metals, such as Cr, Pb, and Cu, in the fecal samples from Wuji Village were significantly lower than those from Chongwei Village, with only the As concentration being significantly higher than that from Chongwei Village (Figure 2b). This may be attributed to factors such as the physiological status of birds and the specific characteristics of toxic metals [7,52]. In general, little egrets in Wuji Village may be at a greater risk of metal contamination than those in Chongwei Village because of their higher metal intake from the foraging environment and diet and lower excretion potential through feces.

### 3.3. Heavy Metals in Feathers and Eggshells of Little Egrets

**Figure 3:**
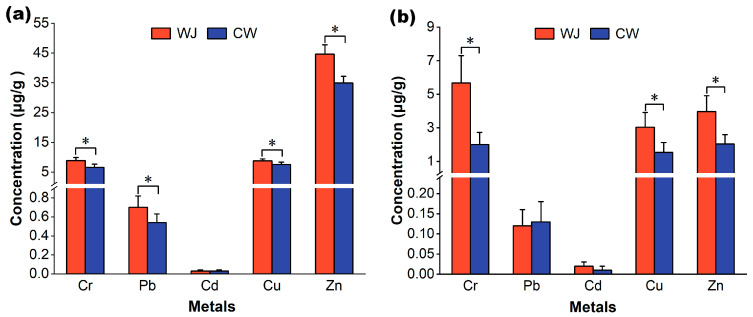
Comparison of metal concentrations measured in feather (**a**) and eggshell (**b**) samples of little egrets from Wuji (WJ) Village and Chongwei (CW) Village heronries. * Significant differences between the two heronries.

The concentrations of heavy metals in feather and eggshell samples of little egrets from the Wuji Village and Chongwei Village heronries are presented in Figure 3. Similar to the foraging environment and food samples, the concentrations of most heavy metals in the feather and eggshell samples of little egrets from Wuji Village were significantly higher than those from Chongwei Village, except for Pb in the eggshell samples and Cd in the feather and eggshell samples (*p* < 0.05) (Figure 3). This difference in heavy metal levels in little egrets from the Wuji and Chongwei Village heronries can be attributed to the habitat-specific difference in the levels of these metals in their diet and foraging environment. These findings are supported by Kim (2010) [53] and Shahbaz (2013) [28], who reported that high metal levels in egret species from Korean and Pakistani colonies were associated with higher levels of these metals in their diet and foraging environments.

As with the food samples, As was not detected in any feather or eggshell samples (Figure 3). This suggests a lack of As accumulation in biological tissues, which may explain the higher As content in the fecal samples of little egrets from Wuji Village compared to those from Chongwei Village. Compared with other metals, Cd exhibited low accumulation in feather and eggshell samples, with BAFs under 0.1 (Figure 3; Table 2). These results are consistent with those of previous studies [13,48]. Although As is widely distributed in nature, As poisoning in wildlife is infrequent due to its detoxification and rapid excretion; thus, it is rarely detected in animals [48]. Previous studies have shown that As ingested by birds is mainly methylated to form non-toxic multi-methylated As metabolites such as dimethylarsinic acid (DMA) and arsenobetaine (AB) in the liver and kidneys, and most of them can be rapidly excreted through urine and feces [54,55]. For instance, a previous study found that zebra finches (*Taeniopygia guttata*) could excrete more than 90% of their total As intake [56]. Although Cd is considered the most toxic metal for living beings among trace metals affecting the reproduction and survival of birds, it exhibits very low intestinal absorption, with less than 7% ingestion in birds [44,57]. Once absorbed, Cd can be chelated with metallothionein (MT) and then transported to the kidneys for storage and excretion [58,59]. Therefore, it is difficult for birds to accumulate Cd, and it cannot be passed to their eggs [48]. We found that the mean Cd concentration (0.03 μg/g) in feather samples of little egrets was much lower than the threshold levels in bird feathers (2 μg/g) associated with adverse effects [28]. Pb is highly toxic to birds; however, only a small amount enters the bloodstream after ingestion, with the majority being sequestered in calcareous tissues [57]. Pb concentrations in the tissues of birds chronically exposed to environmental lead often remain below the threshold levels associated with clinically toxic effects [57]. Notably, although Pb may be effectively transferred and biomagnified (BMF > 1, Table 3), the mean Pb concentration (0.54–0.70 μg/g) found in feather samples of little egrets in the current study was much lower than the toxic threshold of Pb (4 μg/g) in bird feathers [28,60]. Therefore, in the present study, although high concentrations of these toxic metals were found in the foraging environment, they were unlikely to have any adverse effects on the resident little egrets because of their low bioaccumulation potential in bird tissues.

Cu and Zn are essential metals required for the functioning of living organisms and have toxic effects only at high concentrations [12,44]. Although the BAFs and BMFs for Zn and Cu in the feathers and eggshells of little egrets were relatively high (Table 2 and Table 3), the mean concentrations of Cu (feathers: 7.56–8.77 μg/g; eggshells: 1.54–3.04 μg/g) and Zn (feathers: 34.97–44.59 μg/g; eggshells: 2.04–3.97 μg/g) in the current study were lower than those found in other studies [13,19,21,61] and remarkably lower than the threshold level of Zn toxicity in birds (1200 μg/g) [62]. Therefore, the Zn and Cu levels detected in this study do not pose a risk to birds.

Cr is a non-essential element that causes major disturbances to the reproductive health of birds [20,63]. The BMF of Cr in little egret feathers was >1 (Table 3), indicating that Cr can be biomagnified in the food chain. The mean Cr concentrations (6.58–8.86 μg/g) in feather samples of little egrets from Wuji Village and Chongwei Village heronries were higher than those reported in previous studies [13,20,48,64] for different birds, including little egrets. A comparable level (7.45–14.15 μg/g) was found in house crow (*Corvus splendens*) feathers from Punjab, India [65]. Moreover, the levels detected in this study were higher than the toxic Cr threshold (2.8 μg/g) in bird feathers [28]. Although the threshold Cr level in bird eggs has not been established, the mean Cr concentrations (Wuji Village heronry: 5.67 μg/g; Chongwei Village heronry: 2.01 μg/g) in little egret eggshell samples in the current study were comparable to the established toxic threshold in bird feathers. Feathers and eggshells are frequently sampled to assess toxic metal contamination in birds. Feathers are often used as indicators of toxic metals deposited in the blood and body [19,20]. Metal contamination of eggshells can represent egg contamination, which may provide valuable information on the risks posed to reproduction and nestling health [17]. A previous study found that hatching failure in birds may be associated with Cr contamination in eggs [61]. Therefore, together with previous findings, our results suggest that Cr contamination may adversely affect the reproduction and health of little egrets (especially from the Wuji Village heronry) inhabiting rice fields in Hainan.

### 3.4. Reproductive Performance of Little Egrets in Different Heronries

To further verify the differences in the reproductive performance of little egrets in the Wuji Village and Chongwei Village heronries, we assessed several reproductive endpoints of little egrets in both heronries. The results of these reproductive endpoints are shown in Table 4.

Although similar clutch size and fledgling success rates were observed between Wuji Village and Chongwei Village, significantly lower hatching and breeding success rates were observed in Wuji Village than at the less contaminated site (*p* < 0.05) (Table 4). The inferior breeding performance of free-living bird populations may be associated with various factors. For example, a previous study reported that the hatching success of little egrets in Sichuan, Southwest China, is reduced primarily due to human removal of eggs [66]. Adverse weather conditions, such as strong gales, may also negatively affect the breeding success of bird populations by destroying nests and affecting nestling survival [66]. In our study, egg removal by humans was unlikely because local governments and nearby residents have implemented protective measures to minimize human disturbance to breeding populations. Notably, the proportion of destroyed nests was higher in Chongwei Village than in Wuji Village because of gales and rainstorms (Table A1). This may be because, in Chongwei Village, the nests are mainly built on bamboo forests, which are more vulnerable to gales and rainstorms. Therefore, weather has a more obvious negative effect on breeding success in Chongwei than in Wuji. Nevertheless, lower hatching and breeding success rates were observed in Wuji Village than in Chongwei Village, indicating the presence of hidden factors affecting the breeding success of both heronries.

Metal pollution negatively affects bird reproduction. Previous studies have reported lower hatching, fledging, and breeding success rates for bird populations in more-metal-contaminated sites than the less-contaminated sites [21,23]. Moreover, improved breeding performance in pied flycatchers (*Ficedula hypoleuca*) near industrial sites has been observed following a reduction in industrial heavy metal emissions [67]. In this study, feather and eggshell samples from Wuji Village showed higher metal concentrations, especially when the Cr concentration exceeded the toxicity threshold in birds (Figure 3). The impaired breeding performance in Wuji Village was likely associated with heavy metal contamination, especially Cr contamination.

Although birds respond and adapt to changing environments, such as metal pollution, they make trade-offs between self-maintenance and reproduction under limited energy and time conditions [23,68]. Birds exposed to high levels of heavy metals may have fewer resources and/or less energy to allocate to invest in reproduction, affecting egg production, egg quality, incubation, and offspring rearing, which results in less hatching and breeding success and poor nestling health [23]. Eggshell thickness is an important indicator of egg quality and a key factor in determining breeding success [27]. Eggshell thinning is related to hatching failure because thinner eggshells tend to break during incubation [21]. In our study, there was no significant difference in eggshell thickness between the two heronries (Table A2), indicating that eggshell quality is not responsible for the lower hatching and breeding success in Wuji Village. Notably, a previous study showed that eggshell thickness varies greatly in different regions of the egg [27]. This study did not account for that variation in its measurements, which may have obscured potential differences in eggshell thickness between the two heronries. Interestingly, a higher level of Cr, which exceeded the toxicity threshold in birds, was transferred from females (Cr in feathers) to their eggs (Cr in eggshells) in Wuji Village (Figure 3), suggesting that Cr accumulation in eggs may be closely related to hatching failure (embryo mortality) [61]. Notably, the association between Cr contamination and the impaired reproductive performance of little egrets was not statistically confirmed in this study due to the sampling procedure. In addition, higher levels of Cr exposure and risk to nestlings in Wuji Village due to maternal transfer and food resources may only induce sublethal toxic effects, resulting in poor nestling growth and health without obvious fledging failure (Table 4). Therefore, further studies with improved monitoring and sampling efforts are needed to clarify the effects and mechanisms of Cr contamination regarding various aspects of reproduction, including parental investment, nestling growth, and health.

### 3.5. Limitations of the Study and Future Perspectives

The current study had a few limitations that must be considered when interpreting our results, and they need to be carefully analyzed for future research. Firstly, the association between Cr contamination and the impaired reproductive performance of little egrets was not statistically confirmed due to the sampling procedure. Further studies with improved monitoring and sampling efforts are necessary to clarify the link between Cr contamination and various aspects of the reproduction of little egrets in our study area. Secondly, we considered only the metal exposure levels at a specific time of the breeding season. The sampling and subsequent comparison of the metal exposure levels at different times of the breeding season can provide more insights into the risks posed to bird reproduction and health. Finally, different factors, such as weather, habitat differences, anthropogenic stressors, and parasites, should be considered for meaningful interpretation of the results. Finally, future studies should pay more attention to the sublethal effects of metal pollution (Cr) on nestlings in our study area.

## 4. Conclusions

In this study, we demonstrated that the foraging habitats of little egrets on Hainan Island are variously contaminated by heavy metals, with Cd, As, and Cr being the main pollutants that are closely related to agricultural activities, such as the use of chemical fertilizers and pesticides. However, although higher levels of Cd, As, and Pb were found in the foraging habitats, these metal elements exhibited no or negligible bioaccumulation in the feathers and eggshells of little egrets in both the Wuji Village and Chongwei Village heronries, which is unlikely to cause any adverse effects on bird health. Higher levels of heavy metal exposure and risk were found in the feathers and eggshells of little egrets in Wuji Village than in Chongwei Village due to their higher metal intake from the foraging environment and food items and lower excretion potential through feces. Notably, the Cr concentrations in feather and eggshell samples from Wuji Village exceeded the toxic threshold in birds, suggesting that high maternal Cr, associated with toxic effects, was transferred from laying females to their eggs in Wuji Village. Moreover, significantly lower hatching and breeding success rates were observed in Wuji than in Chongwei Village. Although the poor breeding performance of free-living bird populations may be affected by various factors, the impaired breeding performance of little egrets in Wuji Village may be closely related to Cr contamination because Cr enrichment in eggs has been confirmed to cause hatching failure. The results indicate that waterbirds breeding in rice fields are under threat from heavy metal contamination, and feathers and eggshells can be useful indicators of environmental contamination for those metals that are subject to bioaccumulation. Further studies with improved monitoring and sampling efforts are necessary to clarify the potential link between Cr contamination and various aspects of the reproduction of little egrets in our study area.

## Figures and Tables

**Table 1:** Average concentrations of heavy metals in the sediment and water of foraging habitats of different little egret heronries.

Metals	Sediment (μg g^−1^)				Water (μg L^−1^)			
	Chongwei Village	Wuji Village	BV ^a^	TEL ^c^	Chongwei Village	Wuji Village	St ^b^	WHO ^d^
Cr	16.28 ± 7.67	78.28 ± 15.77 *	61.00	37.30	0.74 ± 0.18	2.82 ± 0.81 *	10.00	50.00
Pb	6.84 ± 2.58	11.80 ± 0.98 *	26.00	35.00	4.69 ± 1.51	7.82 ±1.07 *	10.00	10.00
Cd	0.68 ± 0.18	1.36 ± 0.32 *	0.097	0.60	0.07 ± 0.02	0.06 ± 0.03	1.00	3.00
As	0.82 ± 0.30	11.36 ± 4.54 *	11.20	5.90	0.63 ± 0.36	1.82 ± 0.66 *	50.00	10.00
Cu	3.07 ± 0.70	4.62 ± 0.71 *	22.60	35.70	2.33 ± 0.62	4.22 ± 1.22 *	10.00	2000
Zn	9.51 ± 3.30	15.53 ± 1.82 *	74.20	123.00	9.58 ± 1.81	17.66 ± 11.99 *	50.00	3000

^a^ BV: Chinese environmental background value for soil [31]. ^b^ St: Chinese Environmental Quality Standards for Surface Water, Class I Standards. ^c^ TEL: Threshold effect level of Canadian Freshwater Sediment Quality Guidelines [42]. ^d^ WHO: WHO Guidelines for Drinking-Water Quality [43]. * Significant difference between the two heronries at *p* < 0.05.

**Table 2:** Bioaccumulation factors (BAFs) for food, feces, feathers, and eggshells of little egrets.

Metals	Food	Feces	Feathers	Eggshells
Cr	0.109 ± 0.054	0.224 ± 0.171	0.259 ± 0.152	0.103 ± 0.037
Pb	0.038 ± 0.009	0.238 ± 0.119	0.069 ± 0.015	0.014 ± 0.007
Cd	0.024 ± 0.007	0.552 ± 0.199	0.029 ± 0.012	0.011 ± 0.004
As	ND	0.471 ± 0.397	ND	ND
Cu	0.492 ± 0.222	6.301 ± 1.220	2.181 ± 0.340	0.579 ± 0.195
Zn	2.536 ± 0.845	8.980 ± 4.295	3.274 ± 0.453	0.235 ± 0.060

ND: BAF cannot be calculated.

**Table 3:** Biomagnification factors (BMFs) for different tissues of little egrets.

Metals	Feces	Feather	Eggshell
Cr	1.70 ± 0.91	2.18 ± 0.59	0.95 ± 0.31
Pb	6.07 ± 2.66	1.82 ± 0.31	0.37 ± 0.16
Cd	20.27 ± 5.38	0.96 ± 0.30	0.44 ± 0.15
As	ND	ND	ND
Cu	13.21 ± 1.90	4.64 ± 0.83	1.33 ± 0.67
Zn	3.33 ± 0.82	1.34 ± 0.21	0.10 ± 0.04

ND: BMF cannot be calculated.

**Table 4:** Comparison of breeding parameters of little egrets from Wuji Village and Chongwei Village heronries.

Breeding Metric	Mean ± SD (N)		*p*-Value
	Wuji Village	Chongwei Village	
Clutch size	3.82 ± 0.77 (68)	3.88 ± 0.73 (84)	0.640
Hatching success %	0.77 ± 0.20 (68)	0.88 ± 0.16 (84)	<0.001
Fledging success %	0.93 ± 0.13 (68)	0.96 ± 0.11 (84)	0.133
Breeding success %	0.71 ± 0.19 (68)	0.84 ± 0.17 (84)	<0.001

## Data Availability

The data will be made available upon request.

## Appendix A

**Table A1:** Comparison of the number of destroyed nests of little egrets in both heronries.

Sites	Number of Destroyed Nests	Number of Total Nests	Vandalism Rate (%)
Wuji Village	4	72	5.56
Chongwei Village	11	95	11.58

**Table A2:** Comparison of eggshell thickness (mm) of little egrets in both heronries.

Sites	Mean ± SD (N)	*p*-Value
	Min–Max	
Wuji Village	0.18 ± 0.02 (62) 0.12–0.22	0.217
Chongwei Village	0.19 ± 0.01 (59) 0.14–0.21	
